# Analyse von 6581 Fuß- und Sprunggelenkverletzungen einer Notaufnahme im Zeitraum von 2010–2017

**DOI:** 10.1007/s00113-021-01081-9

**Published:** 2021-09-30

**Authors:** Patrick Pflüger, Markus Wurm, Peter Biberthaler, Dominik Pförringer, Moritz Crönlein

**Affiliations:** Klinik und Poliklinik für Unfallchirurgie, Klinikum rechts der Isar, Technische Universität München, München, Deutschland

**Keywords:** Fuß, Sprunggelenk, Epidemiologie, Unfallchirurgie, Osteoporose, Foot, Ankle, Epidemiology, Trauma surgery, Osteoporosis

## Abstract

**Hintergrund:**

Sprunggelenk- und Fußverletzungen gehören zu den häufigsten Krankheitsbildern in der Traumatologie. Ziel dieser Studie war es, die demografischen Veränderungen von Patienten mit Fuß- und Sprunggelenkverletzungen, das zeitliche Auftreten und die Entwicklung über die Jahre in einer Notaufnahme zu untersuchen.

**Material und Methoden:**

Retrospektive Datenauswertung von Patienten, welche aufgrund einer Fuß- und Sprunggelenkverletzung in der Notaufnahme im Zeitraum von 2010 bis 2017 behandelt wurden. Die Patienten wurden mittels ICD-Codes identifiziert und die demografischen Veränderungen, das zeitliche Auftreten und die Entwicklung über die Jahre analysiert. Mittels Quantil-Quantil-Diagramm wurden kontinuierliche Variablen auf Normalverteilung getestet und, falls zutreffend, mittels t‑Test oder nichtparametrischem Mann-Whitney-U-Test auf Signifikanz überprüft.

**Ergebnisse:**

Insgesamt wurden 6581 Fuß- und Sprunggelenkverletzungen in die Analyse eingeschlossen. Das mittlere Alter von Patienten mit einer Fußfraktur war 39 ± 17,4 Jahre und mit einer Sprunggelenkfraktur 47 ± 19,2 Jahre (*p* < 0,001). Ligamentäre Verletzungen des Sprunggelenks traten insbesondere bei jüngeren Patienten in den Sommermonaten auf. Die Altersverteilung bei Sprunggelenkfrakturen zeigte für Männer einen Häufigkeitsgipfel zwischen dem 30. und 39. Lebensjahr und bei Frauen ein vermehrtes Auftreten ab dem 50. Lebensjahr. In der Altersgruppe zwischen 65 und 75 Jahren stellten Trimalleolarfrakturen die zweithäufigste Sprunggelenkfraktur dar. Frakturen des Fußes betrafen am häufigsten Patienten im Alter von 20 bis 29 Jahren, wobei bei Frauen ein 2. Häufigkeitsgipfel zwischen dem 50. und 59. Lebensjahr zu beobachten war.

**Schlussfolgerung:**

Ligamentäre Verletzungen des Sprunggelenks sind häufige Krankheitsbilder in der Notaufnahme und betreffen v. a. jüngere Patienten. Sprunggelenkfrakturen zeigten für Frauen eine bimodale Altersverteilung, und insbesondere Bi- und Trimalleolarfrakturen waren vermehrt bei Frauen ab dem 65. Lebensjahr zu beobachten. Aufgrund der erhöhten Prävalenz von Fuß- und Sprunggelenkfrakturen bei älteren Frauen sollte in diesem Patientenkollektiv eine weitere Osteoporoseabklärung veranlasst werden.

## Abstract (en)

**Background:**

Foot and ankle injuries are among the most common lesions in traumatology, accounting for more than one in ten human fractures. The aim of this study was to investigate the demographic changes of patients with foot and ankle injuries, the temporal occurrence and the development over the years in an emergency department.

**Material and methods:**

Retrospective study of patients treated for foot and ankle injuries in the emergency department from 2010–2017. Patients were identified by ICD codes and demographic changes, temporal occurrence and the development over the years were analyzed. Using quantile-quantile plots, continuous variables were tested for normal distribution and, if applicable, tested for significance using t‑tests or non-parametric Mann-Whitney U‑tests.

**Results:**

A total of 6581 foot and ankle injuries were included in the analysis. The mean age of patients with a foot fracture was 39 ± 17.4 years and with an ankle fracture 47 ± 19.2 years (*p* < 0.001). Ligamentous ankle injuries occurred particularly in younger patients during the summer months. The age distribution of ankle fractures showed a peak incidence between 30–39 years of age for men and an increased occurrence in women over 50 years. In the age group between 65 and 75 years trimalleolar ankle fractures represented the second most common ankle fracture. Fractures of the foot most commonly affected patients aged 20–29 years, with a second peak incidence in women between the ages of 50 and 59 years.

**Conclusion:**

Ligamentous injuries of the ankle joint are common injuries in the emergency department and affect mainly younger patients. Ankle fractures showed a bimodal age distribution for women and especially bimalleolar and trimalleolar fractures were more common in women aged 65 years and older. Because of the increased prevalence of foot and ankle fractures in older women, further osteoporosis evaluation should be initiated in this patient population.

## Einleitung

Sprunggelenk- und Fußverletzungen gehören zu den häufigsten Krankheitsbildern in der Traumatologie. Mehr als jede 10. Fraktur des Menschen entfällt auf diese anatomische Region. In den gegenwärtig publizierten internationalen epidemiologischen Studien zeigt sich eine steigende Inzidenz (neuaufgetretene Fälle in einer Population innerhalb einer bestimmten Zeit) dieser Verletzungen, mit einem zu verzeichnenden Anstieg von instabilen Frakturen in der Altersgruppe über 65 Jahren [13, 36]. Für Deutschland existieren aktuell keine derartigen Daten.

Aufgrund der enormen Herausforderungen für die Versorgungsrealität, die mit einem derartigen Wandel einhergeht, haben wir daher in einer retrospektiven Datenanalyse die demografischen Veränderungen, das zeitliche Auftreten sowie die Entwicklung des Auftretens von Fuß- und Sprunggelenkverletzungen über einem Zeitraum von 8 Jahren analysiert.

## Hintergrund und Fragestellung

Verletzungen des Sprunggelenks und des Fußes gehören zu den häufigsten traumatologischen Krankheitsbildern und sind ursächlich für jede 3. Vorstellung in einer chirurgischen Notaufnahme weltweit [4, 8].

Bezogen auf das Sprunggelenk zeigen sich sowohl für ligamentäre Verletzungen als auch Frakturen steigende Inzidenzen [5, 9, 13, 22]. Während Distorsionen mit ligamentären Verletzungen eher in der jungen Population beobachtet werden [30, 38], variiert das Auftreten von Frakturen hinsichtlich der Altersverteilung mitunter deutlich [13, 34]. In einer Arbeit von Elsoe et al. aus dem Jahr 2018 zeigte sich für Dänemark eine bimodale Altersverteilung der weiblichen Bevölkerung mit einem erneuten Anstieg der Inzidenz um das 70. Lebensjahr [13]. Darüber hinaus konnte eine saisonale Häufigkeitsverteilung mit Inzidenzhöchstwerten in kalten Wintermonaten beobachten werden [13].

Bezogen auf Verletzungen des Fußes zeigen sich in Untersuchungen aus Dänemark und den Niederlanden jährliche Inzidenzraten von 142–226/100.000 Einwohner. Während bei Männern höchste Inzidenzraten um das 20. Lebensjahr beobachtet werden konnten [11, 28], zeigte sich in der weiblichen Bevölkerung analog zu den Sprunggelenkverletzungen eine bimodale Altersverteilung mit einem Anstieg der Inzidenz nach dem 50. Lebensjahr [28].

Die vorhandenen epidemiologische Studien geben jedoch ein sehr inhomogenes Bild wieder, da gewisse Patientenkohorten bzw. Verletzungen nicht eingeschlossen wurden [2, 7–9, 11, 12, 20, 27, 31, 32, 35, 39]. Zudem fehlen aktuelle Daten, da die zuletzt publizierten Studien lediglich einen Zeitraum bis ins Jahr 2014 abbilden [2, 13]. *Nach unserem Wissen* gibt es bis dato keine Studie, welche die epidemiologischen und demografischen Entwicklungen von Fuß- und Sprunggelenkverletzungen in Deutschland untersucht hat.

Ziel dieser Untersuchung war es deshalb, das zeitliche Auftreten, die Entwicklung über die Jahre sowie die demografischen Daten der Patienten mit einer Fuß- und Sprunggelenkverletzung in Deutschland zu analysieren und in einem weiteren Schritt hieraus aktuelle Behandlungsempfehlungen abzuleiten.

## Material und Methoden

Retrospektive Datenanalyse von Patienten, welche im Zeitraum von 2010 bis 2017 in der Notaufnahme des Klinikums rechts der Isar der Technischen Universität München aufgrund einer Fuß- und/oder Sprunggelenkverletzung behandelt wurden.

Die Stadt München zählt mit einer Einwohnerzahl von ca. 1,5 Mio. als drittgrößte Stadt Deutschlands [6]. Die Klinik und Poliklinik für Unfallchirurgie des Klinikums rechts der Isar ist eine von 4 Kliniken der Maximalversorgung in München, welche als überregionales TraumaZentrum DGU® zertifiziert ist. Sie stellt als eine von 10 Kliniken in München die Notfallversorgung für unfallchirurgische Patienten über 24h an 7 Tagen die Woche sicher [10].

Die Datenauswertung erfolgte pseudonymisiert anhand der amtlichen Klassifikation für Diagnosen in der ambulanten und stationären Versorgung in Deutschland (ICD-10-GM). Eine schriftliche Patienteneinwilligung war daher nach Artikel 27 (4) des Bayerischen Krankenhausgesetz nicht notwendig.

Patienten mit den folgenden ICD-10-Codes [26] wurden in die Analyse eingeschlossen: S82.3–S82.9, S86.0, S92.–S92.9, S93.2–8, S93.4–S93.43 und S93.5–6. Es erfolgte eine Unterteilung in Sprunggelenkfrakturen (S82.), Fußfrakturen (S92.), ligamentäre Verletzungen des Sprunggelenks (S93.2, S93.4) und Achillessehnenrupturen (S86.0), Luxationen im Fußbereich (S93.3), Verstauchungen und Zerrungen des Fußes (S93.5–6). Die Sprunggelenkfrakturen wurden entsprechend den ICD-Codes in distale Tibia‑, Fibula- (proximal, Schaft), Innenknöchel‑, Außenknöchel‑, Bi- und Trimalleolarfrakturen subklassifiziert. Bei den Fußfrakturen erfolgte eine Unterteilung in Rückfuß- (Talus- und Kalkaneusfrakturen), Mittelfuß- (Fraktur eines oder mehrerer Fußwurzelknochen) und Vorfußfrakturen (Fraktur der Mittelfußknochen und Zehen). Zudem wurden für die Auswertung das Alter, das Geschlecht und das Behandlungsdatum analysiert.

### Statistische Auswertung

Kontinuierliche Variablen wurden als Mittelwert und Standardabweichung angegeben. Mittels Quantil-Quantil-Diagramm wurden kontinuierliche Variablen auf Normalverteilung getestet *und*, falls zutreffend, mittels t‑Test oder nichtparametrischem Mann-Whitney-U-Test auf Signifikanz überprüft. Bei kategorialen Variablen erfolgte die Angabe von Häufigkeiten bzw. Prozentangaben, und es wurden die „odds ratio“ und das 95 %-Konfidenzintervall berechnet. Die Analyse der Daten erfolgte mittels RStudio (RStudio Team 2020, RStudio: Integrated Development Environment for R. RStudio, PBC, Boston, MA, USA, URL http://www.rstudio.com/).

## Ergebnisse

Insgesamt wurden 6581 Fuß- und Sprunggelenkverletzungen in die Analyse eingeschlossen. Entsprechend der Unterteilung nach ICD-Codes zeigte sich eine leichte Zunahme der Anzahl der Sprunggelenk- und Fußfrakturen sowie der ligamentären Verletzungen des Sprunggelenks zwischen 2010 und 2017 (Tab. 1).

**Table 1:** 

Diagnosen	2010	2011	2012	2013	2014	2015	2016	2017
Fußfrakturen	144	133	178	192	135	213	184	242
Fußverstauchungen/-luxationen	86	70	81	163	121	92	101	95
Lig. Sprunggelenkverletzungen	319	383	398	465	278	549	507	483
Sprunggelenkfrakturen	89	100	137	156	69	144	119	155
Summe	638	686	794	976	603	998	911	975

### Sprunggelenk

**Figure 1:**
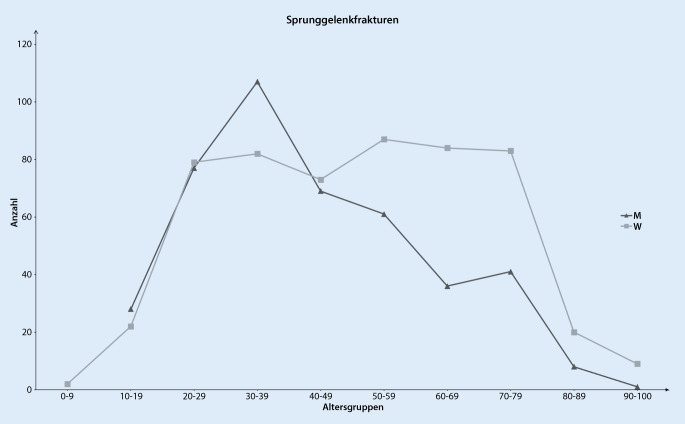

**Figure 2:**
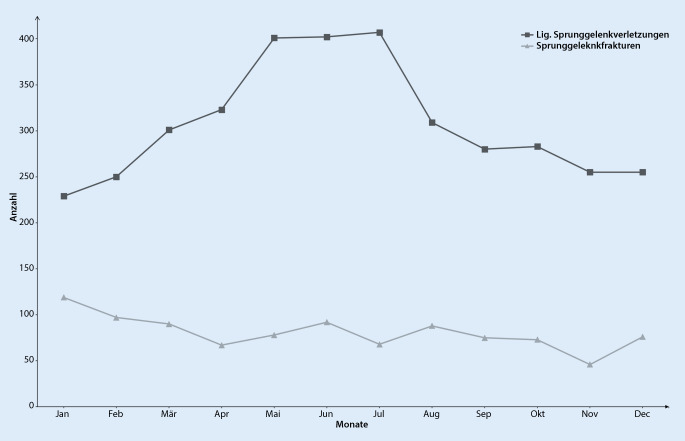

**Figure 3:**
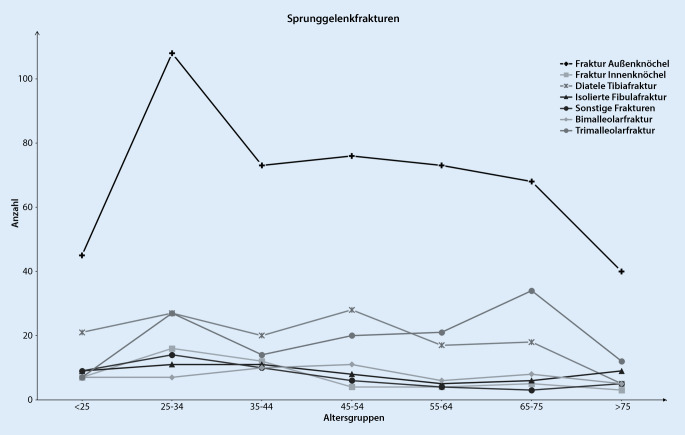

**Figure 4:**
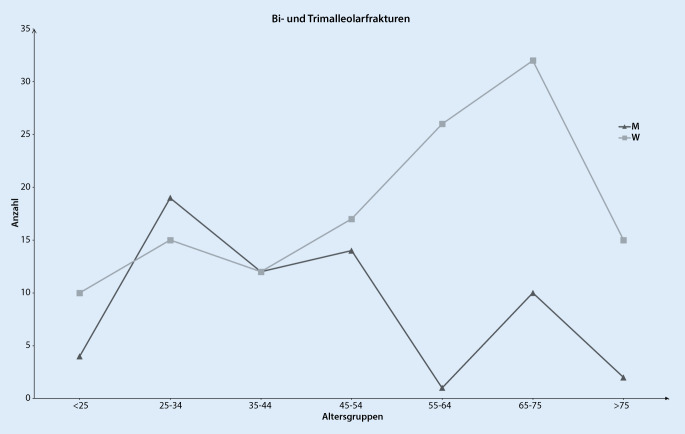

Im genannten Untersuchungszeitraum wurden insgesamt 4824 Sprunggelenkverletzungen in die Analyse einbezogen. Hiervon waren 969 Frakturen des Sprunggelenks, 160 Achillessehnenrupturen und 3695 ligamentäre Verletzungen des Sprunggelenks. Das mittlere Alter der Patienten mit einer Sprunggelenkfraktur war 47 ± 19,2 Jahre mit 44 % Frauen und 56 % Männern. Am häufigsten erlitten Patienten im Alter zwischen 30 und 39 Jahren eine Sprunggelenkfraktur, wobei bei Frauen nach dem 50. Lebensjahr ein erneuter Anstieg festzustellen war (Abb. 1). Betrachtet man das Auftreten aller Sprunggelenkfrakturen, stratifiziert nach Monaten, so zeigten sich ein Häufigkeitsgipfel zu Jahresbeginn sowie ein 2. Anstieg im Juni (Abb. 2). Mit einem Anteil von 50 % aller Sprunggelenkfrakturen war die Fraktur des Außenknöchels am häufigsten, gefolgt von der distalen Tibiafraktur (14 %) und den Trimalleolarfrakturen (14 %) (Tab. 2). Von Frakturen des Außenknöchels waren v. a. Menschen zwischen dem 25. und 34. Lebensjahr betroffen (Abb. 3). Distale Tibiafrakturen hatten ihren Häufigkeitsgipfel zwischen 45 und 54 Jahren und Trimalleolarfrakturen zwischen dem 65. und 74. Lebensjahr (Abb. 3). Betrachtet man nur die als instabil zu wertenden Bi- und Trimalleolarfrakturen, so zeigte sich bei Männern ein Häufigkeitsgipfel in der Altersgruppe von 25 bis 34 Jahren und bei Frauen zwischen dem 65. und 75. Lebensjahr (Abb. 4). Patienten >65 Jahre mit einer Bi- oder Trimalleolarfraktur waren 2,45-mal [KI:1,18–5,06] häufiger weiblich.

**Table 2:** 

Art der Sprunggelenkfraktur	Anzahl	Anteil in %
Fraktur Außenknöchel	483	50
Distale Tibiafraktur	136	14
Trimalleolarfraktur	135	14
Isolierte Fibulafraktur	59	6
Bimalleolarfraktur	54	6
Fraktur des Innenknöchels	51	5
Sonstige Frakturen	51	5
*Total*	969	100

Das mittlere Alter der Patienten mit einer ligamentären Verletzung des Sprunggelenks war 33 ± 15,1 Jahre mit 48 % Frauen und 52 % Männern. Patienten mit einer ligamentären Verletzung des Sprunggelenks waren damit im Vergleich zu Patienten mit einer Sprunggelenkfraktur jünger (*p* < 0,001). Ligamentäre Verletzungen waren sowohl bei Männern als auch Frauen am häufigsten in der Altersgruppe zwischen 25 und 34 Jahren, und mit zunehmendem Alter hat die Anzahl bei beiden Geschlechtern abgenommen. Betrachtet man die Verteilung nach Monaten, so zeigte sich ein vermehrtes Auftreten von ligamentären Verletzungen in den Monaten von Mai bis Juli (Abb. 2).

Patienten mit einer Achillessehnenruptur waren im Mittel 46 ± 16,3 Jahre und in 81 % der Fälle männlich. Am häufigsten waren Patienten im Alter von 35 bis 44 Jahren betroffen.

### Fuß

**Figure 5:**
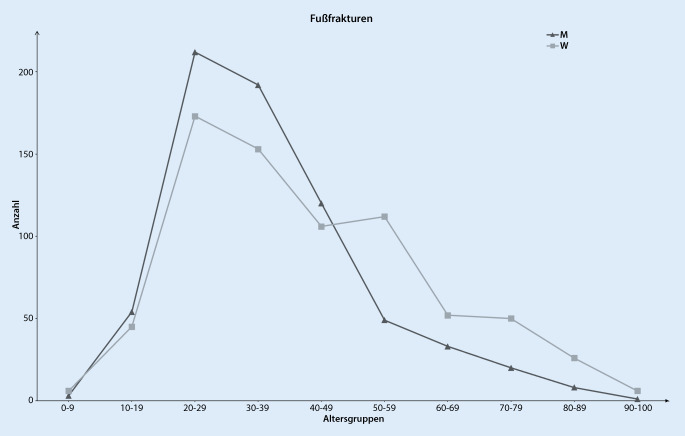

Im Untersuchungszeitraum wurden insgesamt 1757 Fußverletzungen in die Analyse einbezogen. Hiervon waren 1421 Frakturen, 43 Luxationen und 293 Verstauchungen und Zerrungen des Fußes. Das mittlere Alter der Patienten mit einer Fraktur des Fußes war 39 ± 17,4 Jahre und 51 % waren weiblich. Damit waren Patienten mit einer Fußfraktur im Vergleich zu denen mit einer Sprunggelenkfraktur jünger (*p* > 0,001). Am häufigsten erlitten sowohl Männer als auch Frauen in der Altersgruppe zwischen 20 und 29 Jahren eine Fraktur des Fußes (Abb. 5). Bei Frauen war ein erneuter Häufigkeitsgipfel zwischen dem 50. und 59. Lebensjahr zu beobachten. Patienten >55 Jahre mit einer Fußfraktur waren 2,51-mal [KI:1,89–3,34] häufiger weiblich. Betrachtet man das Auftreten aller Fußfrakturen, stratifiziert nach Monaten, so zeigte sich ein Häufigkeitsgipfel im Juli und August. Mit einem Anteil von 45 % aller Fußfrakturen waren die Zehen am häufigsten betroffen, gefolgt von den Mittelfußknochen (32 %) und dem Kalkaneus (11 %) (Tab. 3). Nach Art der Fußfraktur aufgeteilt, zeigte sich eine unimodale Altersverteilung mit einem Häufigkeitsgipfel zwischen dem 25. und 34. Lebensjahr.

**Table 3:** 

Art der Fußfraktur	Anzahl	Anteil in %
Zehen	639	45
Mittelfußknochen	451	32
Kalkaneus	153	11
Fußwurzelknochen	99	7
Talus	68	5
Sonstige	11	1
*Total*	1421	100

Das mittlere Alter der Patienten mit einer Verstauchung und Zerrungen des Fußes war 35 ± 15,3 Jahre und 58 % waren weiblich. Patienten mit einer Verstauchung oder Zerrungen des Fußes waren damit im Vergleich jünger (*p* < 0,001). Verstauchungen und Zerrungen des Fußes waren sowohl bei Männern als auch Frauen am häufigsten in der Altersgruppe zwischen 25 und 34 Jahren, und mit zunehmendem Alter hat die Anzahl bei beiden Geschlechtern abgenommen.

## Diskussion

In dieser Studie wurden erstmals über einen Zeitraum von 8 Jahren in einer Notaufnahme in Deutschland das zeitliche Auftreten, die Entwicklung über die Jahre sowie die demografischen Daten von 6851 Patienten mit einer Fuß- und/oder Sprunggelenkverletzung analysiert.

Es zeigt sich, trotz leichter Schwankungen, eine tendenziell zunehmende Anzahl an Sprunggelenkfrakturen in der von uns untersuchten Patientenklientel. Im Allgemeinen kann dieser Trend auch durch die internationalen Analysen der letzten Jahre bestätigt werden [9, 13]. Diese Beobachtung lässt sich möglicherweise durch eine deutliche Zunahme von Sportverletzungen im jüngeren Patientenkollektiv und die demografische Entwicklung in den letzten Jahren erklären [9, 33]. Bei den Sportverletzungen sind neue Sportarten hinzugekommen, welche ein besonderes Risiko für die untere Extremität darstellen, und jüngere Patienten zeigten eine tendenziell zunehmende körperliche Aktivität [19, 33]. Im älteren Patientenkollektiv ist insbesondere bei Frauen ab dem 50. Lebensjahr eine steigende Prävalenz für Osteoporose und damit ein erhöhtes Frakturrisiko infolge eines inadäquaten Traumas zu beobachten [16, 17].

Hinsichtlich der Häufigkeit des Auftretens einer Sprunggelenkfraktur ergab sich für Männer eine unimodale und für Frauen eine bimodale Altersverteilung. Ein ähnliches Verteilungsmuster konnte auch von Elsoe et al. 2018 in einer retrospektiven Analyse von 9767 Sprunggelenkfrakturen in Dänemark beobachtet werden [13]. Bei den Fußfrakturen zeigte sich eine ähnliche Alters- und Geschlechtsverteilung wie beim Sprunggelenk, jedoch waren die Patienten mit durchschnittlich 39 Jahren etwas jünger. Dies konnte so auch in einer epidemiologischen Studie zu Frakturen in Dänemark und einer Analyse von 5912 Fußfrakturen beobachtet werden [11, 28]. Diese Altersverteilung kann dadurch erklärt werden, dass Fuß- und Sprunggelenkfrakturen bei jüngeren Patienten am häufigsten aufgrund eines Sportunfalls auftreten und diese Verletzungen vornehmlich bei Männern zu beobachten sind [13, 29, 33]. Insbesondere bei Sportarten wie Fußball, Basketball und Football kommt es häufig zu Sprunggelenkverletzungen [38]. Die Tatsache, dass Menschen im Sommer körperlich aktiver sind, kann auch das gehäufte Auftreten von Fußfrakturen im Juli und August erklären [24]. Rasmussen et al. haben diese saisonale Verteilung ebenfalls in einer Studie von 2020 in Dänemark beobachtet [28]. Das vermehrte Auftreten von Fuß- und Sprunggelenkfrakturen bei Frauen ab dem 50. Lebensjahr kann der bis zu 4‑fach erhöhten Prävalenz einer Osteoporose bei Frauen im Vergleich zu Männern zugeschrieben werden [17], zumal in diesem älteren Patientenkollektiv Niedrigenergietraumen die häufigste Ursache für eine Fuß- und Sprunggelenkfraktur sind [13, 27, 28].

Frakturen des Fußes betrafen am häufigsten die Vorfußregion, gefolgt von Frakturen des Rück- und Mittelfußes. Eine vergleichbare Häufigkeitsverteilung auf die 3 unterschiedliche Fußbereiche konnten ebenfalls Shibuya et al. in den USA und Rasmussen et al. in Dänemark beobachten [28, 31].

Bei den Sprunggelenkfrakturen waren Trimalleolarfrakturen nach Außenknöchelfrakturen die zweithäufigste Sprunggelenkfraktur, und insbesondere Frauen zwischen dem 65. und 74. Lebensjahr waren hiervon betroffen. Eben dieser Trend, dass insbesondere die Inzidenz von instabilen Sprunggelenkfrakturen bei Patientinnen über 60 Jahren angestiegen ist, konnte in Untersuchungen von Elsoe et al. in Dänemark und Thur et al. in Schweden beobachtet werden [13, 36]. Eine Eingruppierung der Trimalleolarfrakturen in den Bereich der Fragilitätsfrakturen wird nicht zuletzt aufgrund dieser Beobachtung von einer Reihe von Autoren gefordert [3, 9, 13]. Entsprechend der Definition der Weltgesundheitsorganisation sind Fragilitätsfrakturen die Folge eines inadäquaten Traumas, wie z. B. einem Sturz aus dem Stand oder aus geringer Höhe [16, 25]. Diese beschriebenen Verletzungsmuster sind gerade bei älteren Patienten ursächlich für die instabilen Sprunggelenkfrakturen und unterstützen damit die These, dass Bi- und Trimalleolarfrakturen bei älteren Patienten zu den Fragilitätsfrakturen zu zählen sind [13, 21, 36]. Dies würde bedeuten, dass das Behandlungsmanagement bei diesen Patienten multidisziplinär zwischen Unfallchirurgie, *Geriatrie,* Ergo‑/Physiotherapie und Sozialdienst erfolgen sollte, um den speziellen Anforderungen geriatrischer Patienten gerecht zu werden. Darüber hinaus konnte gezeigt werden, dass bei Patienten mit zunehmendem Alter sowie Vorhandensein von Nebenerkrankungen die Komplikationsrate und die Einjahresmortalität ansteigen [15, 23]. Insbesondere Patienten älter als 65 Jahre haben eine erhöhte Komplikationsrate und benötigen häufiger eine poststationäre Pflegeeinrichtung [1]. Sollte sich diese Entwicklung weiter fortsetzten, so muss darüber diskutiert werden, ob instabile Sprunggelenkfrakturen bei älteren Patienten ebenfalls in die Indikationsgruppen des Kriterienkatalogs AltersTraumaZentrum DGU® aufgenommen werden sollten [37].

Ligamentäre Sprunggelenkverletzung traten im Untersuchungszeitraum häufiger auf als Frakturen des Sprunggelenks. Dies zeigte sich so auch in einer Untersuchung in den Niederlanden, welche eine bis zu 10-mal höhere Inzidenz von Sprunggelenkverstauchungen im Vergleich zu Sprunggelenkfrakturen feststellte [13, 22]. Eine mögliche Erklärung hierfür ist, dass in biomechanischen Untersuchungen gezeigt wurde, dass für eine Fraktur der Fibula im Vergleich zur Außenbandruptur eine etwa 10-mal so große Kraft wirken muss [14, 18]. Darüber hinaus ist zu bedenken, dass Verstauchungen des Sprunggelenks insbesondere bei jüngeren Patienten auftreten und die körperliche Aktivität in den letzten Jahren bei dieser Patientenpopulation zugenommen hat [19, 22]. In unserem Patientenkollektiv zeigte sich sowohl bei Männern als auch bei Frauen eine unimodale Altersverteilung mit Häufigkeitsgipfeln in der Altersgruppe von 25 bis 34 Jahren. Diese Altersverteilung konnten auch Kemler et al. in den Niederlanden und Waterman et al. in den USA bei Patienten mit Sprunggelenkverstauchungen beobachten [22, 38]. Die ligamentären Verletzungen des Sprunggelenks traten in unserer Untersuchung vermehrt in den Sommermonaten auf. Eine mögliche Erklärung hierfür ist, dass Sprunggelenkverstauchungen oftmals die Folge eines Outdoor-Sportunfalls sind und Menschen im Sommer körperlich aktiver sind [24, 33, 38].

## Limitationen

Die Studie weist aufgrund ihres retrospektiven Designs einige Limitationen auf:

- Da die Analyse der Daten anhand der ICD-Codes in einer pseudonymisierten Form erfolgte, können mögliche Fehler bei der Verschlüsselung der ICD-Codes aufgetreten sein.
- Aufgrund fehlender klinischer Daten kann keine Aussage über den Traumamechanismus, die Klassifikation oder relevante Begleiterkrankungen getroffen werden.
- Es wurden nur Patienten, welche in der Notaufnahme behandelt wurden, in die Analyse eingeschlossen.
- Für einige beobachtete Verletzungen zeigte sich eine zu geringe Fallzahl, sodass hieraus keine allgemeingültigen Schlüsse gezogen werden können.

## Fazit für die Praxis

- Ligamentäre Sprunggelenkverletzungen betrafen v. a. jüngere Patienten und waren häufiger als Sprunggelenkfrakturen zu beobachten.
- Die Altersverteilung bei Patienten mit einer Sprunggelenkfraktur zeigte bei Männern um das 30. Lebensjahr und bei Frauen ab dem 50. Lebensjahr Häufigkeitsgipfel.
- Aufgrund des vermehrten Auftretens von Bi- und Trimalleolarfrakturen bei Frauen ab dem 65. Lebensjahr ist zu diskutieren, ob diese instabilen Sprunggelenkfrakturen zu den Fragilitätsfrakturen zu zählen sind.
- Fußfrakturen traten gehäuft bei Patienten zwischen dem 20. und 29. Lebensjahr auf, wobei bei Frauen ein erneuter Anstieg ab dem 50. Lebensjahr zu beobachten war.
- Aufgrund des gehäuften Auftretens von Fuß- und Sprunggelenkfrakturen bei älteren Frauen infolge eines inadäquaten Traumas sollte an eine weitere Abklärung hinsichtlich einer möglichen Osteoporose gedacht werden.
